# Therapeutic Interventions Targeting Beta Amyloid Pathogenesis in an Aging Dog Model

**DOI:** 10.2174/157015911798376217

**Published:** 2011-12

**Authors:** Sarah B Martin, Amy L.S Dowling, Elizabeth Head

**Affiliations:** a Sanders Brown Center on Aging, University of Kentucky, Lexington KY, USA; b Department of Molecular and Biomedical Pharmacology, University of Kentucky, Lexington KY, USA

**Keywords:** Alzheimer's disease, canine, statins, BACE-1 inhibitors, metal-chelators, A-beta vaccination.

## Abstract

Aged dogs and humans share complex cognitive and pathological responses to aging. Specifically, dogs develop Alzheimer’s Disease (AD) like beta-amyloid (Aβ) that are associated with cognitive deficits. Currently, therapeutic approaches to prevent AD are targeted towards reduced production, aggregation and increased clearance of Aβ. The current review discusses cognition and neuropathology of the aging canine model and how it has and continues to be useful in further understanding the safety and efficacy of potential AD prevention therapies targeting Aβ.

## INTRODUCTION

Alzheimer’s disease (AD) is the most common cause of dementia in the elderly and currently affects 5.5 million people in the United States alone. AD is characterized by the presence of senile plaques and neurofibrillary tangles (NFTs) in the brain [1-3]. Given that the greatest risk factor for developing AD is age and that our elderly population is rapidly rising, it is critical to identify preventative measures and early interventions to slow or stop the disease [4]. The current review provides an overview of how the dog model of human aging and AD has been and continues to be a beneficial preclinical model to test the efficacy and safety of certain types of disease modifying treatments for AD.

### Beta-Amyloid (Aβ)

Senile plaques, which are primarily composed of β-amyloid (Aβ), are one of two neuropathological characteristics of AD [5]. The Aβ peptide is produced by the sequential cleavage of the amyloid precursor protein (APP) by β- and γ- secretases [6, 7]. Cleavage by γ-secretase results in differing lengths of Aβ, with the 42 amino acid form, Aβ_42,_ making up most of the insoluble deposits found in the AD brain [8]. Aβ is largely thought to play a role in the disease process because it is a unifying pathological feature of the genetically diverse forms of AD.

The gene encoding APP is located on chromosome 21. Because three copies of this chromosome are present in individuals with Down Syndrome (DS), DS research has been particularly important in elucidating the role of Aβ in AD [9, 10]. Individuals with DS show Aβ deposition as early as 8 years of age [11] and have classical neuropathological features of AD by their early forties [12-15]. The localization of the APP gene to chromosome 21 initiated a search for genetic linkages in families with autosomal dominant AD [7], leading to the association of missense mutations in the APP gene with familial AD [16-19]. Furthermore, duplication of the APP gene causes autosomal dominant AD in the absence of DS [20]. Past studies of DS and familial AD have established that Aβ is a pathological feature of both disorders and that Aβ production and decreased clearance [21, 22] is at the root of disease progression. The importance of Aβ in the pathogenesis of AD has led to the search for therapeutic interventions targeting Aβ.

### Animal Models for Aβ Therapeutics: Beyond the Mouse

Potential AD therapeutics are generally screened first in transgenic mouse models of AD. As well as being inexpensive and easy to house, mouse models are ideal because they age quickly and their entire genome is mapped. However, mice do not produce human sequence Aβ naturally [23]. Transgenic mouse models of AD are produced by over expressing mutant human APP alone or combined with transgenic presenilin 1 (PS1) and presenilin2 (PS2) genes in mice, which leads to Aβ plaque formation [24]. Importantly, unlike humans, transgenic mice show cellular and behavioral resilience to Aβ pathology and thus do not develop the extensive neuronal loss observed in the AD brain. While transgenic mouse models continue to get more complex and more closely replicate human AD pathology, there are several components of the disease process that mice cannot model. For example, most AD patients neither have the genetic mutations present in transgenic mice nor overexpress these mutations if present at such high levels. While transgenic mouse models have been and will continue to be instrumental in the development of therapeutics, the basic evolutionary differences between the two species makes taking potential therapeutics from mice to humans less direct.

The successful development of therapeutic interventions targeting Aβ benefits from testing in animal models that naturally recapitulate critical aspects of human disease. Diseases associated with brain aging are especially challenging, due to the time required to track both the aging process and the interplay between pathology and cognition. An ideal animal model has several key features in addition to the natural development of AD pathology. For example, it is beneficial for an animal model of AD to have a naturally diverse response to aging, given that humans show individual variations with age [25-27]. While some people show mild cognitive decline with age, others develop severe cognitive decline. Still others develop no visible signs of decline, the definition of “successful aging”. This spectrum of cognition in aging is suggested to result from both genetic and environmental factors [28, 29], another key feature of an ideal animal model of AD. Although increased variability presents statistical challenges, requiring additional animals and experimental studies, natural diversity offers certain unique advantages. The ability to compare similarly aged animals with differing cognitive functions allows researchers to distinguish pathological underpinnings associated with cognitive function.

Not all cognitive domains are equally vulnerable to aging or AD in humans. For example, the first sign of AD in humans, often referred to as amnestic mild cognitive impairment (MCI), is defined by a gradual decline in episodic memory function with preserved general cognitive and functional abilities, combined with no evidence of dementia [27, 30]. As the disease progresses into clinical AD, patients show a disturbance in at least one of the following cognitive functions: language, motor skills, visual processing or executive function [31]. More specifically, AD patients show a decline in declarative learning, while procedural abilities remain intact. Animal models that capture these cognitive characteristics are well suited to develop and test therapeutic interventions to translate into human clinical trials in AD patients. Unfortunately, no single animal model can replicate all aspects of AD, but each can provide unique strengths that advance our knowledge. 

## CANINE MODEL OF AGING

The canine model has a rich literature in psychological and neurobiological research, dating back to the 1800s. Canines are useful for aging research, have moderate lifespans of 12 to 20 years, depending on the breed [32, 33], and are easy to handle due to a long history of domestication [34]. Furthermore, canines are highly motivated to perform consistently on cognitive tests using simple food rewards, making food deprivation paradigms unnecessary. In contrast, mice are not readily cooperative in performing behavioral tasks, so physiological stressors including food restriction, water deprivation and immersion in water are often used [35]. Therefore measures of cognition may be engaging other processes involved in stress response, confounding performance scores. Importantly, the cognitive decline and progressive age-associated neuropathology observed in dogs parallels that of humans.

### Cognition and Aging in Dogs

Cognitive testing procedures for canines were initially developed by modifying non-human primate cognitive tests [36]. A variety of tests have been developed to assess cognitive function in various domains and corresponding brain localization in dogs. Table **1** [originally published in 37] outlines cognitive domains assessed in dog aging and how they compare with assessment tasks for non-human primate and humans. Many of these tests are analogous to cognitive tests used for nonhuman primates and humans. As in humans, canine individual variability and domain-specific cognitive vulnerabilities are key features of decline with age [36]. Beginning in middle age, individual variability in cognitive scores begins to increase, with the largest variability seen in aged dogs [38]. Additionally, vulnerability to decline with age varies as a function of cognitive domain and the cortical circuits engaged. For example, size discrimination learning is sensitive to age, while simple object discrimination is not [36, 38, 39], similar to monkey models of aging [40-44]. Further, prefrontal dependent reversal learning is more age sensitive than discrimination learning [36, 38, 39].

Efficacy of a therapeutic intervention for AD is ultimately measured by its ability to stabilize or improve function in cognitive domains compromised by AD. The canine parallels features of human cognitive decline and has well established measures that are domain specific [reviewed in 37]. Use of the canine model allows researchers to study the complex cognitive implications of therapeutic interventions, an important measure preceding clinical trials.

### Neuropathology in Aging Dogs

In addition to the cognitive features of aging, dogs naturally develop brain changes similar to human brain aging [reviewed in 37]. Importantly, a key feature that makes dogs useful as a model of human Aβ pathogenesis was the observation in 1956, by Braumühl, who reported “Alzheimer’s-like” senile plaques in aged dogs [reviewed by 34]. Dogs initially develop plaques between the ages of 8 and 9 years [37, 45], a relatively young age, compared to many non-human primate models with naturally occurring AD-like pathology [46, 47]. Deposition of human-like Aβ accumulation in the aged canine brain has been well-described [34, 48-52]. There are significant homologies between canines and humans in the APP protein sequence, as well as in many enzymes involved in the processing of APP to form Aβ (http://www.ensembl.org/Canis_familiaris/). 

Diffuse plaques are the predominant subtype of Aβ in the canine model [49, 52-56], whereas more compact neuritic plaques (i.e. Aβ associated with tau positive neurites) are predominant in AD Fig. (**1A**, **B**). However, evidence suggests that diffuse plaques are extensive in humans and appear early in AD progression [57, 58]. As observed in humans, Aβ pathology in canines shows specific brain regions being differentially vulnerable to Aβ [1, 2, 50, 52, 59-61]. Modeling early stages of AD is critical, given that AD therapies are likely to be most effective early in disease progression. Importantly, unlike humans, canines do not develop NFTs [47, 53, 55, 56], the second neuropathological characteristic of AD. However, the lack of NFTs in canines is a feature of the model that can be used to differentiate between the effects of Aβ and NFTs in the disease process, an ongoing subject of AD research.

Both humans and canines develop cerebrovascular amyloid angiopathy (CAA), the deposition of Aβ in association with the cerebrovasculature Fig. (**1C**, **D**). The presence of CAA in canines enhances the utility of this model in developing and testing therapeutic interventions to target Aβ. In both canines and humans with CAA, the blood vessels of the brain typically contain the shorter, 40 amino acid-long species of Aβ [62-64]. Further, the distribution of CAA is similar in both species, with the occipital cortex being predominantly susceptible. Vascular Aβ may compromise the blood-brain barrier, disrupt vessel wall viability [65] and cause microhemorrhages [66, 67].

Recently, the focus in AD pathogenesis has shifted from Aβ plaques to consider smaller, soluble forms of Aβ assemblies called Aβ oligomers. Oligomers are highly toxic and impair synaptic function [68]. Furthermore, elevated oligomer levels are strongly associated with cognitive dysfunction [69, 70]. A recent study by Pop *et al.*, examined the accumulation of oligomeric Aβ in the temporal lobe of canines. These results provide evidence that canines, like humans, experience an increase in toxic oligomers with age [71].

Another key feature of AD that is also found in dogs is neuronal loss in the presence of cognitive deficits. A study by Pugliese *et al.,* (2007) demonstrated that cognitive deficits correlated with loss of purkinje cells [72]. More recently, a study by Insua *et al.,* (2010) examined neuroadrenergic neurons in the locus ceruleus of aged canines, a group of neurons that are vulnerable to AD in humans [73, 74]. They found that cognitively impaired dogs exhibited significant reduction in noradrenergic neurons [75]. 

The efficacy of Aβ modifying therapies are measured by neuropathological and cognitive improvement. Studies have found an association between Aβ load and cognitive dysfunction in aging dogs, similar to humans with AD [48]. As with humans, there is not a perfect link between the extent of Aβ pathology and the severity of cognitive decline, suggesting that other neuropathological cascades are involved in neurodegeneration. The natural presence of cognitive decline and Aβ neuropathology make the dog a valuable model in developing therapeutics for AD. 

## POTENTIAL TARGETS FOR AΒ MODIFICATION

Aβ deposition is thought to result from one or more of the following mechanisms: 1) increased production of Aβ, 2) decreased Aβ clearance and/or 3) enhanced aggregation of Aβ Fig. (**2**). Strategies to decrease Aβ deposition have focused on modulating all of these mechanisms individually. For example, studies focused on preventing Aβ production inhibit the enzymatic step required to cleave APP into an Aβ peptide. However, the monomeric Aβ peptide alone is easily cleared from the brain *via *the blood-brain barrier and is not considered a toxic form of Aβ [76]. The Aβ peptide becomes more toxic as it aggregates to the oligomer structural state [77-81] and subsequently further assembles into insoluble fibrils [reviewed in 82]. Therefore, a considerable amount of research is also focused on therapeutic interventions that prevent Aβ aggregation or enhance clearance of Aβ and Aβ aggregates. Here we will review some promising therapies that target production (antioxidant diet, behavioral enrichment, BACE1 inhibition, statins), aggregation (metal chelators) and clearance (Aβ immunotherapy) of Aβ Fig. (**2**). The dog model has been or may be useful for determining the efficacy of each potential therapy.

### Antioxidants, Behavioral Enrichment and Prevention of Aβ Deposition

Lifestyle factors are poorly understood, yet are potentially effective at preventing Aβ production. Specifically, nutrition and lifestyle enrichment (e.g. exercise, education and social interaction) effectively prevent cognitive decline and Aβ production [83]. Results from mouse models of AD suggest that an antioxidant enriched diet [84, 85] and/or environmental enrichment [86] decreases production of Aβ peptides. Human studies have determined that nutrition and other lifestyle factors offer cognitive benefits and reduce the risk of developing AD [87-93]. However, the genetic and environmental variability of the human population makes it difficult to determine the efficacy of these complex interventions and their mechanisms of action. 

To measure cognitive and neurobiological benefits of lifestyle factors in aged dogs, diet and environment have been evaluated singularly and in combination, using a variety of learning and memory tasks. In the first study, aged beagles were treated with either a standard senior dog diet or an antioxidant-rich senior dog diet for 6 months [88, 94]. The formulation differences between control and enriched diets were as follows: D,L-alpha-tocopherol acetate (120 ppm vs. 1050 ppm), L-carnitine (<20ppm vs. 260ppm), D,L-alpha-lipoic acid (<20ppm vs. 128ppm), ascorbic acid as Stay-C (<30ppm vs. 80ppm) and 1% inclusion of each of the following (1 to 1 exchange for corn): spinach flakes, tomato pomace, grape pomace, carrot granules and citrus pulp. Oddity discrimination learning was assessed after 6 months of treatment. Aged dogs fed an antioxidant-enriched diet had significantly less age-dependent cognitive impairment than aged dogs fed the control diet. 

In the follow up study, cognitive benefits of the antioxidant diet were evaluated after two years of treatment in the aged dogs [95]. The antioxidant-enriched diet was as described above. Further, the cognitive effects of the anti-oxidant diet either alone or in combination with behavioral enrichment were measured. Behavioral enrichment consisted of group exercise for 15 minute intervals twice a week, exposure to toys that were alternated weekly and housing with kennel mates. After two years of treatment, results suggested an attenuation of age-related cognitive decline that was particularly striking with the combined treatment of behavioral and antioxidant enrichment. At the end of the study, Aβ neuropathology was measured in the prefrontal cortex and the extent of Aβ was significantly decreased in animals receiving the antioxidant enrichment [96]. Further, Aβ pathology was most robustly decreased in animals that received both antioxidant and behavioral enrichment, suggesting that the combination of treatments may affect the production and/or clearance of Aβ. An increase in alpha-secretase enzyme activity in aged dogs treated with the antioxidant and behavioral enrichment protocols, suggests a shift towards non-amyloidogenic APP processing. These canine studies provide support for the combination of antioxidant and behavioral enrichment during the aging process in order to maintain cognition and inhibit Aβ production. Further, these results emphasize the importance of therapeutic interventions that target α-secretase activity. The canine model has lead to an increased understanding of the mechanisms and efficacy of these interventions on cognition and pathology.

### BACE1 Inhibition and Prevention of Aβ Production

Two sequential enzymatic cleavages of APP are necessary to produce Aβ. The first is cleavage of APP by β-secretase APP-cleaving enzyme 1 (BACE1). BACE1 cleaves APP, generating the N-terminus of Aβ. In AD brains, levels of BACE1 protein and activity are approximately twice that of controls [97-99], indicating enhanced production of Aβ. BACE1 is a promising target for the development of therapeutic interventions to lower Aβ production. However, it is important to note that BACE1 has substrates other than APP [100] and toxic side effects could result when BACE1 is fully inhibited. One possible side effect identified in a BACE1 knockout mouse (*BACE1^-/-^*) involved NRG1, a substrate for BACE1 and a protein implicated in schizophrenia [101]. *BACE1^-/-^*mice exhibit endophenotypes typically seen in schizophrenia [101, 102], leading researchers to test whether partial BACE1 inhibition decreases Aβ load while limiting unwanted side effects. Using a heterozygous mouse (*BACE1^+/-^*) to impart partial BACE1 inhibition, McConlogue *et al., *found decreased Aβ burden in 13 and 18 month old *BACE1^+/-^*APP transgenic mice [103]. Thus, BACE1 inhibition remains a potential therapeutic target.

The use of mouse models in designing BACE1 inhibitors has been instrumental. When inhibiting an enzyme that plays critical physiological roles in brain chemistry, it is increasingly important to test the putative therapy in several different models of AD prior to clinical trial. Humans and canines share a 98% homology in BACE1 (http://www.ensembl.org/Canis_familiaris/). Prior to clinical trials, testing therapeutic interventions that target BACE1 inhibition in a dog model may provide additional information regarding the dose requirements, safety and efficacy of these AD therapies.

### Statins, Cholesterol and the Prevention of Aβ Production

Several cross-sectional or case-control epidemiological studies have revealed a striking link between cholesterol-lowering drugs (e.g. statins and others) and a 20-70% reduction in risk of developing AD [104-111]. However some [112-114], but not all [115], prospective studies have reported no link between statin use and protection against dementia. Differential reports of the positive effects of statins on the development of AD may be due to the cohort studied, confounds by indication, type of statin used, age group studied, and type of study conducted (e.g. cross-sectional, case-control or prospective study) [116, 117]. Further, in preliminary AD clinical trials with simvastatin [118] and atorvastatin [119-122], modest cognitive benefits have been reported. In particular, AD patients with mild to moderate dementia who were treated with 80 mg/day atorvastatin had significantly improved scores on one measure of cognition (ADAS-Cog) at 6 months of treatment, with smaller non-significant benefits at 12 months [120]. 

Recent studies strongly suggest once the signs of AD are evident that the use of statins might reflect a preventative approach rather than a treatment. For example, several studies suggest that mid-life cholesterol levels and statin use impact the risk of developing AD [123, 124]. Further, in a 5-year prospective study of individuals who were not demented at the start of the trial, statin use resulted in a 50% reduction in the risk of developing either dementia or cognitive impairments without dementia (CIND), as compared to placebo/control [125]. Thus, modulating mid-life cholesterol and/or statin administration and/or implementing statin use prior to CIND or dementia may be more beneficial than treatment approaches in patients with AD. Prevention studies are long, costly and challenging to conduct, but can be greatly facilitated by appropriate preclinical testing. Studies in animal models can provide preliminary information regarding potential benefits of statins in the prevention of cognitive decline and AD neuropathology.

Statins may reduce the risk of incident AD through the prevention of Aβ production [126, 127]. In rodent models, treatment with inhibitors of 3-hydroxy-3-methylglutaryl coenzyme A (HMG-CoA) or statins reduces Aβ [128]. However, rodents respond to statin treatment by massively upregulating HMG-CoA reductase levels [129-132]. To compensate, long-term studies in rodent often employ physiologically excessive doses, making it difficult to translate the results of these studies into human trials. 

In contrast to rodent models, the dog model is a useful model for studies of chronic statin treatment, given similarities with humans in terms of dose requirements, responsiveness, drug handling and metabolism [129, 133]. For example, in a study of 12 animals, dogs were treated with 80 mg/day of atorvastatin for 14.5 months (Head, unpublished data). Peripheral levels of cholesterol, low density lipoproteins, triglycerides and high density lipoproteins were reduced in treated dogs. Surprisingly, a transient impairment in reversal learning was observed, suggesting prefrontal dysfunction. Spatial memory remained unchanged up to over a year of treatment. The lack of cognitive benefits of treatment was also reflected by a lack of reduction in plasma, cerebrospinal fluid and brain Aβ. Interestingly, BACE1 protein level was decreased in the brains of atorvastatin-treated dogs. This intriguing outcome may suggest that statins might be more useful to prevent the production of Aβ through lowering BACE1 if started in animals in middle age, consistent with human studies indicating that middle-aged individuals using statins are protected from AD. Aged dogs are a unique model that may provide novel insights and translational data to predict outcomes of statin use in human clinical trials.

### Metal Chelators and Reducing Aβ Aggregation

Preventing Aβ aggregation may also be a promising approach to AD prevention. Metal ions such as zinc (Zn^2+)^, Copper (Cu^2+)^ and iron (Fe^3+^) rapidly induce Aβ aggregation [134-137]. Further, Fe^3+^, Zn^2+^ and Cu^2+^ abnormally accumulate in the brains of AD patients [138]. Accumulation of these metals increases with disease progression, and high levels are found in Aβ plaques [139]. Additionally, studies suggest that the presence of Cu^2+^ is required for beta-amyloid aggregation and neurotoxicity [140]. A promising therapeutic approach to AD prevention involves the administration of agents to chelate metal ions and remove them from the blood and brain, which may result in Aβ disaggregation and clearance. For example, in a study of transgenic AD mice (Tg2576), the Cu^2+^ and Zn^2+^chelator clioquinol reduced metal ion accumulation in the brain, reduced Aβ burden by 49% [141] and improved cognitive performance [142]. The use of clioquinol as a treatment for AD has gone to phase II clinical trials [143], with inconclusive results [144, 145]. Following clioquinol administration, patients with mild AD had decreased plasma Aβ_42_ with no cognitive benefit, while patients with severe AD showed cognitive benefit with no decrease in plasma Aβ_42_. Questions of efficacy and safety remain unanswered, in part due to the small sample size and incomplete understanding of the mechanism of clioquinol in decreasing Aβ.

Metal ion chelators have potential in AD therapy, but remain controversial. Manipulating essential metals in the CNS can have neurotoxic effects and it is important to examine the efficacy and pathological mechanism of a potential therapy in more than one animal model prior to clinical trials. Additionally, it is important to test chelation therapies in an animal model that naturally exhibits Aβ pathology, Aβ associated cognitive decline and metal dyshomeostasis with age, in order to offer insight into the mechanisms, efficacy and safety of these interventions. Importantly, dogs exhibit these characteristics and have increased metal ions in the brain with age [146].

### Aβ Vaccination and Increased Clearance of Aβ

As mentioned previously, another approach to reducing Aβ and potential effects on cognition is to clear pre-existing or new deposits. In transgenic mouse models of AD, deposition of Aβ was prevented or significantly reduced by vaccination with fibrillar Aβ_1-42 _[147-150]. In parallel with Aβ reduction, learning and memory was improved by either active [148, 149, 151] or passive immunization [152-155]. 

Aged dogs may be very useful for assessing Aβ-targeted immunization therapies. Using an active immunization approach, dogs were injected with fibrillar Aβ_1-42_ formulated with aluminum hydroxide, an adjuvant safe for use in humans [156]. Over a 2 year period of monthly vaccinations, surprisingly little cognitive improvement was observed on multiple measures of learning and memory, using tasks that are both age and intervention-sensitive. Interestingly, after 22 months of treatment, a significant improvement on reversal learning in treated animals was observed. Overall, error scores in control dogs increased over time, reflecting both an increase in task difficulty and the aging process. However, error scores were differentially affected by treatment, given that animals immunized with fibrillar Aβ showed maintenance of reversal learning ability over time, suggesting maintained frontal lobe function. These results suggest that immunization with fibrillar Aβ_1-42_ leads to improved and maintained executive function in aged dogs, when both pre-existing Aβ and cognitive deficits are typically present**. **At the end of the study, Aβ plaque accumulation, soluble and insoluble Aβ_1-40_ and Aβ_1-42_ were measured. Reductions in Aβ were observed in multiple brain regions that are essential for learning and memory, including the prefrontal, parietal, occipital and entorhinal cortex. 

Studies in higher mammalian species with disease characteristics that naturally parallel those in human AD may be a critically important step in the process of determining whether a drug should be taken to a clinical trial. For example, the promise of Aβ vaccination was derived from research in the transgenic mouse models of AD and rapidly translated into a human clinical trial. However, the clinical trial in patients with AD using a similar formulation of immunizing with fibrillar Aβ_1-42_ (AN1792 study) lead to an unexpected adverse event and an early halt to the study [157], which was not predicted from the mouse studies. Further, the clinical outcomes were not as robust as observed in transgenic mice. In the Swiss cohort of the trial, function was maintained on a global test of cognition (Mini-Mental State Examination) and on a hippocampal-dependent task (visual paired associated test) in vaccinated individuals who developed antibodies capable of binding to plaques [158]. However, in a second, larger study and in contrast to the predicted outcomes based on work in transgenic mice, no differences were observed on several cognitive and disability scales between treated and untreated patients [159]. One promising outcome was that 12 months after treatment, the composite score from a battery of neuropsychological tests indicated less severe memory decline [159]. Eight patients enrolled in the AN1792 study have been autopsied and show Aβplaque reduction without any effect on the extent of NFTs or CAA [160-162]. Interestingly, in a case report by Masliah *et al.,* (2005), the frontal cortex showed the largest response to immunotherapy [162]. Notably, the decreased Aβ pathology persisted 5 years after the last vaccination [163], although reduced brain Aβ did not slow AD progression and 7 these patients had severe end stage dementia prior to death. These findings parallel observations in the dog vaccination study, further emphasizing the need to test therapeutics in natural models of aging and AD. 

Although there were several important differences between the dog and human vaccination studies [reviewed in 156]), dog studies suggest that reducing plaque accumulation or total Aβ in dogs with pre-existing pathology may be insufficient to restore neuronal function without directly targeting neuron health. Evidence from the previously discussed antioxidant and behavior enrichment study suggest alternative pathways that might be used in combination with Aβ vaccination to improve neuronal health. For example, significant improvements in cognition may be achieved by combining Aβ immunotherapy with either behavioral enrichment or an antioxidant diet to restore neuron health after Aβ removal.

## SUMMARY

AD is a complex disease that remains a challenge in terms of developing therapeutics for clinical trials. To date, no disease pathology modifying therapies are commercially available for AD. Although mice and other rodent models are invaluable in learning about the mechanistic pathways involved in AD pathogenesis and identifying therapeutic targets, these studies should be extended to natural models to design safe and effective therapeutic strategies.

Because AD affects multiple pathways, therapeutic strategies may need to target the disease using parallel approaches. As discussed above, this can be achieved by combining therapeutics. For example, it may be beneficial to pair therapies that increase Aβ clearance with those that repair neuron health and attenuate oxidative stress. The canine model complements other animal models of AD and continues to be a beneficial system in which to test the efficacy and safety of therapeutics or preventative approaches for AD.

## Figures and Tables

**Fig. (1):**
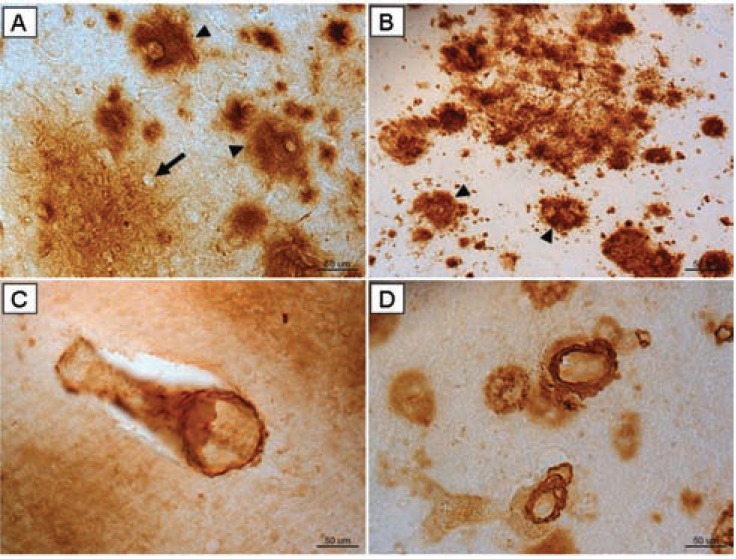
Immunoreactivity for Aβ 1-42 in frontal cortex brain tissue of (**A**) an aged canine and (**B**) an aged human. Compact Aβ deposits are similar in humans and canines (arrow head). The outline of an intact neuron enveloped by a diffuse plaque is visible (arrow). Aβ 1-40 immunoreactivity of cerebral amyloid angiopathy is similar in aged canine occipital cortex (**C**) and aged human occipital cortex (**D**).

**Fig. (2):**
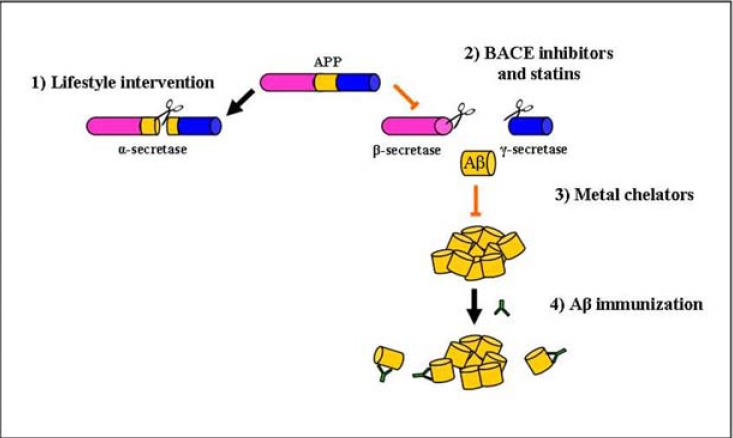
Aβ-modifying therapies can target the modulation of production and clearance. **1**) Lifestyle interventions (e.g. nutrition and lifestyle enrichment) increase α-secretase activity. α-secretase cleaves APP in the middle of the Aβ region (denoted in yellow), releasing a nonamyloidogenic, soluble peptide. **2**) BACE inhibitors and statins decrease β-secretase activity or BACE1 protein levels, which may result in decreased production of Aβ from APP. **3**) Metal chelators prevent metal ions from inducing Aβ aggregation, thereby allowing Aβ peptides to be cleared from the brain. **4**) Immunization therapies use anti-amyloid antibodies to bind and remove Aβ deposits.

**Table 1.** Cognitive Domains Assessed in Dog Aging and Comparison with Nonhuman Primate Tasks and Analogous Tasks Used in Human Neuropsychological Testing

Cognitive Domain	Dog Task	Localization in Dog Brain	Nonhuman Primate Tasks	Examples of Human Neuropsychological Tasks**
Learning	Visual discrimination learning	Medial temporal lobe/parietal lobe*	Visual discrimination learning [40, 164]	digit copy, rotary pursuit, face discrimination [165], object discrimination [166, 167]
	Reward and object approach learning	Nigrostriatal and motor cortex*	Food pickup task, fine motor learning [168, 169]	
Memory	delayed nonmatching to sample acquisition	Rhinal cortex [170]	Object recognition memory task [171]	Delayed recognition and recall, digit span [172]
	delayed nonmatching to sample memory	Rhinal cortex [170]	Object recognition memory task [171]	
	spatial delayed nonmatch to sample acquisition	Dorsolateral prefrontal cortex [170]	Delayed Response Task [43,173]	
	spatial delayed nonmatch to sample memory	Hippocampus [175]	Delayed Response Task [43, 175]	
Executive Function	Visual reversal learning	Prefrontal cortex/medial temporal lobe [175]	Visual reversal learning [40,164]	card or object sorting tasks, set shifting, response inhibition [176]
	Oddity discrimination	Prefrontal cortex/medial temporal lobe*	N/A	
	Egocentric spatial reversal learning	Hippocampal/prefrontal cortex*	Spatial reversal [164]	
	Size concept learning	Prefrontal cortex/medial temporal lobe*	Hierarchical/Relational learning [177]	
Visuospatial Function	Landmark discrimination	Prefrontal cortex/parietal cortex*	Landmark discrimination [178]	Visual construction, block design, spatial learning [166, 167]
	Egocentric spatial learning	Hippocampus/medial temporal lobe*	Spatial learning [164]	

* Proposed localization – not confirmed in lesion studies in dogs

** Neuropsychological tasks for humans that assess function in similar cognitive domains.
